# Preconception vitamin D intake and obstetric outcomes in women using assisted reproductive technology: the Japan Environment and Children’s Study

**DOI:** 10.1186/s12884-022-04861-2

**Published:** 2022-07-05

**Authors:** Hyo Kyozuka, Tsuyoshi Murata, Toma Fukuda, Karin Imaizumi, Akiko Yamaguchi, Shun Yasuda, Daisuke Suzuki, Akiko Sato, Yuka Ogata, Mitsuaki Hosoya, Seiji Yasumura, Koichi Hashimoto, Hidekazu Nishigori, Keiya Fujimori, the Japan Environment, Children’s Study (JECS) Group, Michihiro Kamijima, Shin Yamazaki, Yukihiro Ohya, Reiko Kishi, Nobuo Yaegashi, Koichi Hashimoto, Chisato Mori, Shuichi Ito, Zentaro Yamagata, Hidekuni Inadera, Takeo Nakayama, Hiroyasu Iso, Masayuki Shima, Youichi Kurozawa, Narufumi Suganuma, Koichi Kusuhara, Takahiko Katoh

**Affiliations:** 1 Department of Obstetrics and Gynecology, Fukushima Medical University School of Medicine, 1 Hikarigaoka, Fukushima, 960-1295 Japan; 2 Fukushima Regional Center for the Japan Environmental and Children’s Study, 1 Hikarigaoka, Fukushima, 960-1295 Japan; 3 Fukushima Medical Center for Children and Women, Fukushima Medical University, 1 Hikarigaoka, Fukushima, 960-1295 Japan; 4 Department of Pediatrics, Fukushima Medical University School of Medicine, 1 Hikarigaoka, Fukushima, 960-1295 Japan; 5 Department of Public Health, Fukushima Medical University School of Medicine, 1 Hikarigaoka, Fukushima, 960-1295 Japan

**Keywords:** Preconception care, Pregnancy, Premature birth, Gestational age, Vitamin D

## Abstract

**Background:**

In reproductive medicine, vitamin D (VitD) is of particular interest because its deficiency has been linked to various infertility issues. Thus, preconception care, including appropriate VitD supplementation, is essential, especially in women using assisted reproductive technology (ART). Despite the therapeutic benefits of VitD, adverse events due to a high daily intake may influence obstetric outcomes. However, the effects of either low or high preconception VitD intake on obstetric outcomes, including the outcomes in women who used ART, remain unclear. Therefore, the aim of this study was to examine the association between pre-pregnancy daily VitD intake and obstetric outcomes in Japanese women, including those who conceived through ART.

**Methods:**

Data were obtained from the Japan Environment and Children’s study database comprising 92,571 women recruited between January 2011 and March 2014 in Japan. Participants were categorized into five quintiles according to pre-pregnancy VitD intake (Q1 and Q5 had the lowest and highest VitD intake, respectively) and stratified according to the use of ART. Multiple logistic regression was performed to identify the effects of pre-pregnancy VitD intake on preterm birth (PTB), low-birth weight infant (LBW), and small for gestational age (SGA).

**Results:**

Using Q3 (middle VitD intake) as a reference, our analysis revealed that Q5 (highest VitD intake) showed an increased risk of LBW < 1500 g (adjusted odds ratio [aOR]: 1.09, 95% confidence interval [CI]: 1.00–1.18) and SGA (aOR: 1.26, 95% CI: 1.14–1.39) among women who conceived without ART. Among women who conceived with ART, we found that Q5 (highest VitD intake) showed an increased risk of PTB at < 37 weeks (aOR: 2.05, 95% CI: 1.27–3.31).

**Conclusion:**

The present study revealed that higher VitD intake before pregnancy may affect perinatal outcomes, particularly in women using ART. Our findings may facilitate personalized preconceptional counseling regarding VitD intake based on the method of conception, especially among women using ART.

## Background

Vitamin D (VitD) is an essential secosteroid hormone known for its physiological function in maintaining bone metabolism and health [1]. VitD insufficiency has been associated with the increased occurrence of a variety of cancers and autoimmune diseases. During the coronavirus pandemic, research has been focused on VitD supplementation and its potentially protective role against the risk of respiratory tract infections and other diseases [2].

In reproductive medicine, there is major interest in VitD because its deficiency has been associated with various infertility issues, such as polycystic ovarian syndrome, endometriosis, myoma-induced infertility, male infertility, premature ovarian failure, anti-Mullerian hormone production, steroidogenesis and ovarian folliculogenesis, endometrial receptivity and implantation, and poor prognosis in in vitro fertilization (IVF) (via its crucial role in hypothalamic-hypophyseal system regulation) [3–5]. Therefore, women using assisted reproductive technology (ART) should receive preconception care, particularly appropriate VitD supplementation.

Although its therapeutic benefit is established, adverse events due to a high daily intake may influence obstetric outcomes. Increased intake of VitD supplements in the general population and a growing number of therapeutic prescriptions of VitD (including very high doses) without proper medical monitoring may result in VitD toxicity, a common cause of hypercalcemia [6].

Recommendations for VitD intake during pregnancy vary among the guidelines, primarily owing to regional alimentary habits and environmental variations [7]. The International Federation of Gynecology and Obstetrics and the National Institute for Health and Care Excellence recommend VitD supplementation of 10 μg/day for all pregnant women, the Royal College of Physicians of Ireland recommends 5 μg/day, and the Academy of Nutrition and Dietetics recommends 15 μg/day in pregnant women with VitD deficiency [7]. In Japan, the Ministry of Health, Labour and Welfare recommends a VitD daily adequate intake of 8.5 and 5.5 μg for pregnant women and those of reproductive age (18–49 years), respectively. They also recommend a VitD tolerable upper intake level (UL) of 100 μg/day among reproductive-age women because of its potential toxicity [8].

To the best of our knowledge, no studies have evaluated the effects of either lower or higher preconception VitD intake on obstetric outcomes using a large sample size. Furthermore, preconception VitD intake is essential, particularly among women aiming to conceive by ART. Hence, we aimed to examine whether maternal pre-pregnancy VitD intake is associated with obstetric outcomes such as preterm birth (PTB) and low-birth weight (LBW) in Japanese women, using data from a large Japanese cohort study. This analysis was also stratified according to the use of ART.

## Methods

### Study design

The data used in the study were obtained from the Japan Environment and Children’s Study (JECS). The JECS is an ongoing nationwide, multicenter, prospective birth cohort study funded by the Ministry of the Environment, Japan [9, 10]. The JECS investigated the effects of several environmental factors on children’s health outcomes [9]. A general population of 103,060 pregnancies with 104,062 fetuses was enrolled in the JECS in 15 Regional Centers, covering a wide geographical area in Japan, between January 2011 and March 2014. Follow-up is planned until the children are 13 years old to measure the effect of environmental factors on children’s health [9]. The detailed methodology has been previously reported [9] [10]

### Data collection

At the time of recruitment (during the first trimester of pregnancy), participants were asked to complete a baseline self-administered questionnaire. Follow-up questionnaire surveys were conducted during the second/third trimester and at one month and 6 months after giving birth. Here, we excluded cases with insufficient data and multifetal pregnancies.

### Determination of pre-pregnancy VitD usage, obstetric outcomes, and confounding factors

VitD intake before conception was determined using the food frequency questionnaire (FFQ), completed during the first trimester. Participants were asked for the consumption frequency of various types of food, from the year before pregnancy to their first trimester. These data were used to establish dietary patterns during the preconception period. A standard portion size was specified for each item of the FFQ [10]. Response options for the intake frequency ranged from “almost never” to “ ≥ 7 times/day” for foods such as fish, mushrooms, and eggs, and from “almost never” to “ ≥ 10 glasses/day” for beverages such as milk. The intake frequencies were then multiplied by the specified portion size. The nutrient content of each food was obtained from the Japanese Food Consumption Table, revised 5th edition [11], and the daily VitD intake was the sum of the contents of all food items after being multiplied by the consumption frequency. The FFQ has been validated as a self-administrated dietary questionnaire for the Japanese general population because it is structured according to the eating habits of the Japanese population [12].

Obstetric outcomes included PTB, LBW, small for gestational age (SGA), and hypertension disorders of pregnancy (HDP). PTB before 37 weeks inclusive and PTB before 34 weeks. LBW was classified into two categories: LBW < 2500 g inclusive and LBW < 1500 g [13]. SGA was defined as a birth weight <  − 1.5 standard deviation and corrected for gestational age and sex according to the “New Japanese neonatal anthropometric charts for gestational age at birth” [14, 15]. HDP was defined as the new onset of hypertension (≥ 140/90 mmHg) after conception [10]. ART pregnancy was defined as conception with IVF and/or intracytoplasmic sperm injection (ICSI) or after using cryopreserved, frozen, or blastocyst embryo transfers [15]. Chronic hypertension was defined as the presence of maternal hypertension before pregnancy [10]. Glucose intolerance in JECS was defined as the presence of diabetes mellitus (either insulin-dependent diabetes mellitus or non-insulin-dependent diabetes mellitus) at the time of pregnancy, HbA1c ≥ 6.5% in the first trimester, and/or any steroid use during pregnancy[16, 17]. We considered the following confounding factors: maternal age, body mass index (BMI) before pregnancy, maternal smoking status, maternal educational status, endometriosis, and parity. Maternal age at delivery was categorized into four groups: < 20 years, 20–29 years, 30–39 years, and ≥ 40 years. Pre-pregnancy maternal BMI was calculated by dividing the body weight (kg) by the square of the mother’s height (m). BMI was categorized as follow; < 18.5, 18.5–25.0, or ≥ 25.0 kg/m^2^. The self-reported questionnaire during the first trimester had the following options regarding maternal smoking status: “None,” “Previously I did. But quit prior to current pregnancy,” “Previously I did. But I quit after/during this pregnancy,” and “Currently smoking.” Women who chose “Currently smoking” were considered as the smoking category; otherwise, they were considered non-smokers. Maternal education was categorized into four groups: junior high school, < 10 years of education; high school, 10–12 years; technical/vocational college, 13–16 years; and graduate school, ≥ 17 years. Participants were also requested to answer the following question regarding endometriosis: “Have you ever been diagnosed with endometriosis before pregnancy?” The participants who answered “Yes” were defined as “presence of endometriosis” [18]. Participants were categorized as primiparous or multiparous. Daily energy intake was calculated using the FFQ, completed during the first half of the pregnancy. The inclusion criteria of confounding factors for this study were determined based on clinical importance related to present obstetric complications [13, 15, 18, 19].

### Statistical analyses

The participants were categorized into VitD-intake quintiles (Q1 had the lowest VitD intake, and Q5 had the highest VitD intake) after excluding the cases with multifetal gestation. Maternal characteristics were summarized according to the quintile. Kruskal–Wallis (one-way analysis of variance) and chi-squared tests were used to compare continuous and categorical variables, respectively. Mantel–Haenszel's chi-square test for linear trends was used to analyze the trends in categorical variables. Participants were divided into two groups based on whether they conceived with or without ART. The adjusted odds ratios (aORs) and 95% confidence intervals (CIs) for PTB at < 37 weeks, PTB at < 34 weeks, LBW < 2500 g, LBW < 1500 g, SGA, and HDP were calculated using multiple logistic regression modeling after excluding the cases of chronic hypertension and glucose intolerance. Maternal age, maternal pre-pregnancy BMI, maternal education, maternal smoking status, endometriosis, parity, and daily calorie intake were used to calculate the aOR. This was accomplished by using dummy variables for categorical variables with more than three categories. Statistical analyses were performed using SPSS version 26 (IBM Corp., Armonk, NY, USA) software. A *P*-value < 0.05 was considered indicative of statistical significance.

## Results

**Fig. 1:**
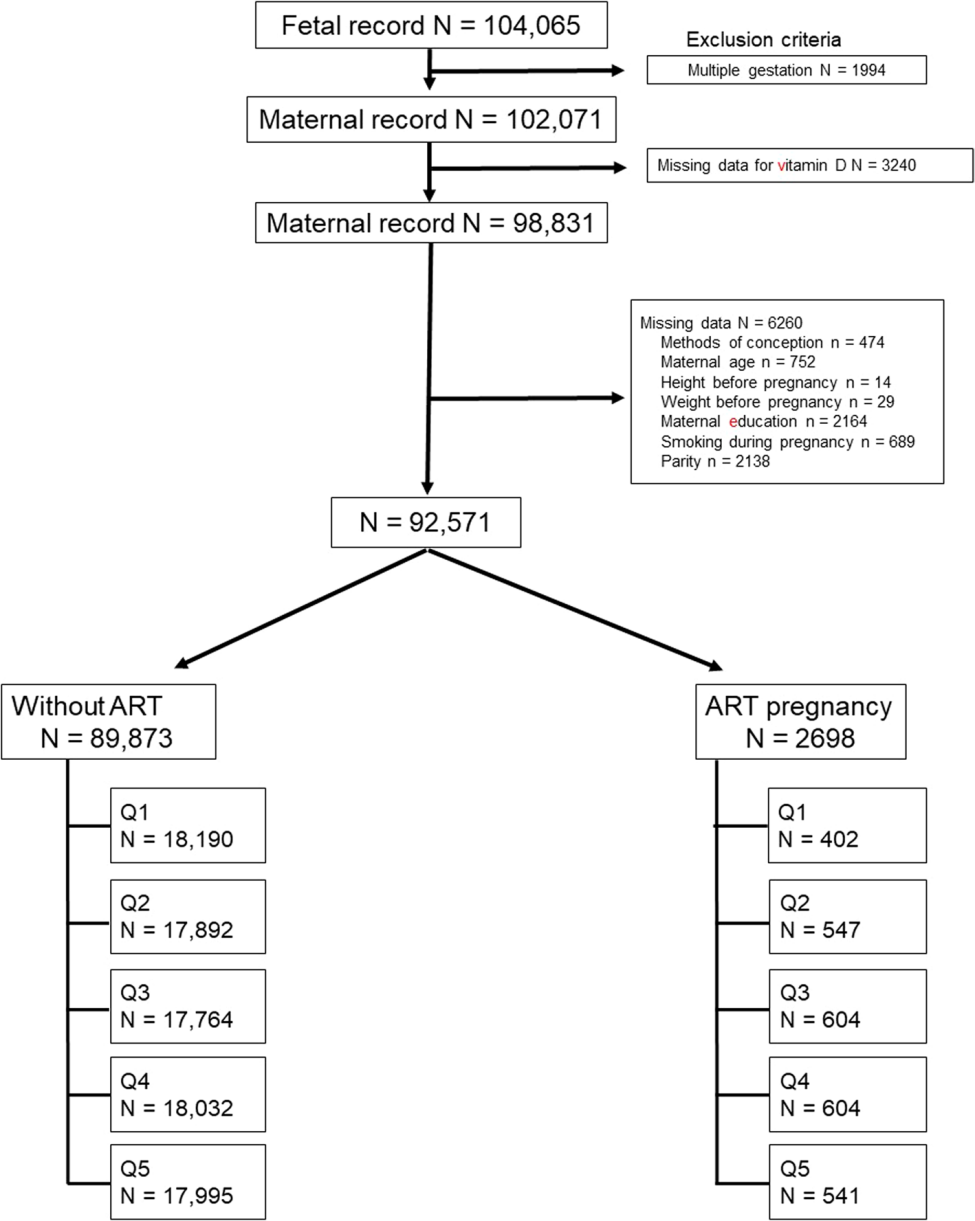
Study flow chart. ART: Assisted reproductive technology

The total number of fetal records in the JECS between 2011 and 2014 was 104,065. Of these, 1994 infants were excluded owing to multifetal gestations. Subsequently, 9500 participants were excluded because of missing data. Thus, 92,571 women met our inclusion criteria, were categorized into quintiles based on the VitD intake, and divided into two groups according to conception with or without ART (Fig. 1).

Table 1 summarizes the maternal medical background parameters and obstetric outcomes according to the pre-pregnancy VitD-intake quintile. The median (interquartile range) pre-pregnancy VitD intake (μg/day) of all participants was 4.3 (2.6–6.7). The median (interquartile range) pre-pregnancy VitD intake (μg/day) of each quintile from Q1 to Q5 was 1.4 (0.8–1.8), 3.0 (2.6–3.3), 4.3 (4.0–4.7), 6.0 (5.5–6.7), and 10.2 (8.6–12.8), respectively.

**Table 1:** Maternal medical background and obstetric outcomes

**Variable**	**Quintile for VitD**					
	**Q1 (low)**	**Q2**	**Q3**	**Q4**	**Q5 (high)**	
	***n* = 18,592**	***n* = 18,439**	***n* = 18,368**	***n* = 18,636**	***n* = 18,536**	***P*-value**
**Maternal medical background**						
VitD intake before pregnancy, μg/day, median (IQR)	1.4 (0.8–1.8)	3.0 (2.6–3.3)	4.3 (4.0–4.7)	6.0 (5.5–6.7)	10.2 (8.6–12.8)	< 0.001^a^
Maternal age, years, mean (SD)	30.0 (5.3)	31.0 (5.0)	31.4 (4.9)	31.8 (4.8)	31.8 (4.9)	< 0.001^b^
Advanced maternal age, %						
< 20	1.6	0.7	0.5	0.5	0.6	< 0.001^c^
20–29	45.7	37.7	35.1	31.6	31.8	
30–39	49.1	57.4	59.6	62.8	62.3	
> 40	3.6	4.1	4.8	5.2	5.3	
BMI before pregnancy (kg/m^2^), %						
< 18.5	17.2	16.6	15.8	15.7	15.1	< 0.001^c^
18.5–25.0	71.7	73.1	74.0	73.9	73.1	
> 25.0	11.1	10.3	10.2	10.4	11.8	
Smoking during pregnancy, %	6.6	4.7	4.3	3.9	4.6	< 0.001^c^
Primipara, %	51.3	42.5	39.2	35.8	32.7	< 0.001^c^
ART pregnancy, %	2.2	3.0	3.3	3.2	2.9	< 0.001^c^
Chronic Hypertension, %	1.3	1.1	1.1	1.2	1.3	0.240
Glucose intolerance, %	1.6	1.7	1.8	1.6	2.1	0.002
Endometriosis, %	3.0	3.6	3.8	3.8	3.9	< 0.001^c^
Maternal education, years, %						
< 10	7.1	4.6	4.1	3.8	4.5	< 0.001^c^
10–12	38.6	32.9	29.6	27.5	29.1	
13–16	38.7	41.8	43.1	42.9	44.2	
> 17	15.7	20.8	23.2	25.8	22.2	
Annual household income, %						
< 2,000,000 JPY	8.7	5.4	4.5	4.2	5.6	< 0.001^c^
2,000,000–5,999,999 JPY	69.6	68.7	67.7	66.1	66.0	
6,000,000–9,999,999 JPY	18.5	21.9	23.4	24.7	23.7	
> 10,000,000 JPY	3.3	3.9	4.5	4.9	4.7	
Daily calorie intake, kcal/day, median (IQR)	1307 (1049–1939)	1523 (1282–1813)	1657 (1416–1952)	1881 (1594–2637)	2254 (1850–3578)	< 0.001^a^
**Obstetric outcomes**						
PTB at < 37 weeks, %	4.5	4.8	4.5	4.4	4.8	0.768^d^
PTB at < 34 weeks, %	0.9	1.0	0.9	0.8	1.0	0.676^d^
LBW < 2500 g, %	8.2	8.3	7.8	7.5	8.2	0.175^d^
LBW < 1500 g, %	0.6	0.6	0.5	0.5	0.6	0.443^d^
SGA, %	5.1	5.0	4.6	5.0	5.6	0.031^d^
HDP, %	3.3	3.1	2.9	3.1	3.1	0.360^d^

*VitD* Vitamin D, *n* Number, *IQR* Interquartile range, *SD* Standard deviation, *BMI* Body mass index, *ART* Assisted reproductive technology, *JPY* Japanese Yen, *PTB* Preterm birth, *LBW* Low-birth weight infant, *SGA* Small for gestational age, *HDP* Hypertensive disorder of pregnancy

^a^ Kruskal–Wallis analysis

^b^ One-way analysis of variance

^c^ Chi-square test

^d^ Mantel–Haenszel’s chi-square test for linear trends

Significant differences were observed among the quintiles in mean maternal age (*P* < 0.001), maternal age category (*P* < 0.001), maternal education (*P* < 0.001), BMI before pregnancy (*P* < 0.001), smoking during pregnancy (*P* < 0.001), rate of primipara (*P* < 0.001), ART pregnancy (*P* < 0.001), glucose intolerance (*P* = 0.002), endometriosis (*P* < 0.001), and annual household income (*P* < 0.001). The rates of maternal age < 20 years, BMI < 18.5 kg/m^2^, smoking, primipara, and maternal education < 10 years were the highest in Q1. Conversely, maternal age of > 40 years, BMI > 25.0 kg/m^2^, and endometriosis were the highest in Q5. The rate of ART pregnancy was the highest in Q3 (3.3%) and the lowest in Q1 (2.2%). The median daily calorie intake was significantly different between the quintiles (*P* < 0.001) with the lowest in Q1 and the highest in Q5. With respect to obstetric outcomes, no significant differences were identified in the occurrence of PTB at < 37 (*P* = 0.768) and < 34 weeks (*P* = 0.676), LBW < 2500 g (*P* = 0.175), LBW < 1500 g (*P* = 0.443), and HDP (*P* = 0.360) along with preconceptional VitD intake. The occurrence of SGA was significantly increased along with VitD intake (*P* = 0.031).

Table 2 depicts the relationship between VitD intake and obstetric outcomes among women who conceived without ART, using Q3 as a reference. Twenty-five thousand five hundred seventy-eight cases were excluded because of the presence of chronic hypertension and glucose intolerance. The risk of SGA increased along with VitD intake (*p* = 0.025). Logistic regression analysis revealed that Q5 showed an increased risk of LBW < 2500 g (aOR: 1.09, 95% CI: 1.00–1.18) and SGA (aOR: 1.26, 95% CI: 1.14–1.39).

**Table 2:** Relationship between VitD intake and obstetric outcomes among women who conceived without ART

	**Quintile for VitD**					
	**Q1 (low)**	**Q2**	**Q3**	**Q4**	**Q5 (high)**	***p*-value**^**a**^
	***n* = 17,687**	***n* = 17,407**	***n* = 17,254**	***n* = 17,532**	***n* = 17,415**	
VitD intake before pregnancy, μg/day, median (IQR)	1.4 (0.8–1.8)	3.0 (2.6–3.3)	4.3 (4.0–4.7)	6.0 (5.5–6.7)	10.2 (8.7–12.8)	
PTB at < 37 weeks						
Number, n	729	784	724	704	749	
Occurrence, %	4.1	4.5	4.2	4.0	4.3	0.812
aOR (95% CI)	0.97 (0.89–1.09)	1.08 (0.97–1.12)	1 (Ref)	0.95 (0.85–1.05)	1.02 (0.92–1.13)	
PTB at < 34 weeks						
Number, n	145	157	138	119	145	
Occurrence, %	0.8	0.9	0.8	0.7	0.8	0.361
aOR (95% CI)	1.02 (0.81–1.30)	1.14 (0.90–1.43)	1 (Ref)	0.85 (0.66–1.08)	1.05 (0.83–1.33)	
LBW < 2500 g						
Number, n	1371	1397	1264	1256	1352	
Occurrence, %	7.8	8.0	7.3	7.2	7.8	0.198
aOR (95% CI)	1.00 (0.92–1.09)	1.09 (1.01–1.18)	1 (Ref)	0.99 (0.91–1.07)	1.09 (1.00–1.18)	
LBW < 1500 g						
Number, n	79	91	68	66	73	
Occurrence, %	0.4	0.5	0.4	0.4	0.4	0.203
aOR (95% CI)	1.17 (0.84–1.63)	1.35 (0.98–1.87)	1 (Ref)	0.97 (0.69–1.37)	1.12 (0.80–1.56)	
SGA						
Number, n	868	860	777	851	963	
Occurrence, %	4.9	5.0	4.5	4.9	5.6	0.025
aOR (95% CI)	1.06 (0.96–1.17)	1.09 (0.99–1.21)	1 (Ref)	1.10 (0.99–1.22)	1.26 (1.14–1.39)	
HDP						
Number, n	415	394	357	377	364	
Occurrence, %	2.4	2.3	2.1	2.2	2.1	0.076
aOR (95% CI)	1.05 (0.91–1.22)	1.06 (0.92–1.23)	1 (Ref)	1.04 (0.90–1.21)	1.00 (0.86–1.16)	

*VitD* Vitamin D, *ART* Assisted reproductive technology, *IQR* Interquartile range, *PTB* Preterm birth, *aOR* Adjusted odds ratio, *CI* Confidence interval, *Ref* Reference, *LBW* Low-birth weight infant, *SGA* Small for gestational age, *HDP* Hypertensive disorder of pregnancy

aOR was calculated by logistic regression analysis, using maternal age (20–29 years old as the reference), body mass index before pregnancy, maternal smoking status, maternal education, endometriosis, parity, and daily calorie intake

^a^ Mantel–Haenszel’s chi-square test for linear trends

Table 3 depicts the relationship between VitD intake and obstetric outcomes among women who conceived with ART, using Q3 as a reference. There is no significant trend in the risk for any obstetric outcome along with VitD intake. As per the logistic regression analysis, Q5 demonstrated an increased risk of PTB at < 37 weeks (aOR: 2.05, 95% CI: 1.27–3.31).

**Table 3:** Relationship between VitD intake and obstetric outcomes among women who conceived with ART

	**Quintile for VitD**					
	**Q1 (low)**	**Q2**	**Q3**	**Q4**	**Q5 (high)**	***p*-value**^**a**^
	***n* = 383**	***n* = 530**	***n* = 578**	***n* = 587**	***n* = 516**	
VitD intake before pregnancy, μg/day, median (IQR)	1.5 (1.0–1.9)	3.0 (2.6–3.3)	4.3 (4.0–4.7)	6.0 (5.4–6.6)	10.0 (8.5–12.1)	
PTB at < 37 weeks						
Number, n	30	35	29	40	50	
Occurrence, %	7.8	6,6	5.0	6.8	9.7	0.206
aOR (95% CI)	1.64 (0.96–2.78)	1.32 (0.79–2.20)	1 (Ref)	1.41 (0.86–2.30)	2.05 (1.27–3.31)	
PTB at < 34 weeks						
Number, n	7	9	7	14	13	
Occurrence, %	1.8	1.7	1.2	2.4	2.5	0.251
aOR (95% CI)	1.50 (0.52–4.34)	1.37 (0.50–3.72)	1 (Ref)	2.03 (0.81–5.09)	1.90 (0.74–4.87)	
LBW < 2500 g						
Number, n	45	50	52	50	57	
Occurrence, %	11.7	9.4	9.0	8.5	11.1	0.727
aOR (95% CI)	1.30 (0.85–2.00)	1.04 (0.69–1.57)	1 (Ref)	0.97 (0.65–1.46)	1.27 (0.85–1.89)	
LBW < 1500 g						
Number, n	8	7	5	8	9	
Occurrence, %	2.1	1.3	0.9	1.4	1.8	0.850
aOR (95% CI)	2.36 (0.76–7.33)	1.45 (0.45–4.64)	1 (Ref)	1.63 (0.53–5.04)	1.79 (0.58–5.54)	
SGA						
Number, n	26	28	32	32	20	
Occurrence, %	6.8	5.3	5.5	5.5	3.9	0.105
aOR (95% CI)	1.21 (0.70–2.09)	0.94 (0.55–1.60)	1 (Ref)	1.01 (0.61–1.68)	0.65 (0.36–1.17)	
HDP						
Number, n	19	28	27	31	24	
Occurrence, %	5.0	5.3	4.7	5.3	4.7	0.841
aOR (95% CI)	1.01 (0.55–1.84)	1.00 (0.57–1.74)	1 (Ref)	1.16 (0.68–1.97)	0.93 (0.53–1.65)	

*VitD* Vitamin D, *ART* Assisted reproductive technology, *IQR* Interquartile range, *PTB* Preterm birth, *aOR* Adjusted odds ratio, *CI* Confidence interval, *Ref* Reference, *LBW* Low-birth weight infant, *SGA* Small for gestational age, *HDP* Hypertensive disorder of pregnancy

aOR was calculated by logistic regression analysis, using maternal age (20–29 years as reference), body mass index before pregnancy, maternal smoking status, maternal education, endometriosis, parity, and daily calorie intake

^a^ Mantel–Haenszel’s chi-square test for linear trends

## Discussion

To the best of our knowledge, this is the first study to examine the association between VitD intake before pregnancy and adverse obstetric outcomes based on the use of ART, using the largest Japanese birth cohort dataset. First, we found that the median (interquartile range) pre-pregnancy daily VitD intake among Japanese women was 4.3 (2.6–6.7) μg/day. Second, we found a relationship between basic maternal characteristics and daily VitD intake before pregnancy. The lowest daily VitD-intake group tended to be socioeconomically disadvantaged, i.e., maternal age < 20 years, smoking during pregnancy, lower maternal education level, and lower annual household income. Conversely, daily calorie intake increased along with daily VitD intake. Third, we found that excessive daily VitD intake could inversely increase some perinatal outcomes. Among all participants, the risk of SGA increased along with the daily VitD intake category. When we focused on women conceived by ART, although there was no dose-dependent relationship, the highest VitD-intake group (Q5) was associated with PTB at < 37 weeks, considering the middle VitD-intake group (Q3) as a reference.

VitD deficiency is a public concern worldwide. However, VitD is a fat-soluble vitamin and may accumulate in the body tissues, leading to toxicity. Therefore, we examined the association between higher VitD intake before pregnancy and obstetric outcomes; this is the first report which shows the adverse outcomes of higher pre-pregnancy VitD intake in women using ART.

VitD has important functional biological implications related to calcium, glucose, immune homeostasis, and anti-inflammation [20–23]. Most prior studies have concentrated on a deficiency in daily VitD intake and its association with adverse maternal and fetal outcomes, such as gestational diabetes mellitus, preeclampsia (a symptom of HDP, SGA, and PTB), and recurrent miscarriage [24]. VitD supplementation is easily administered without apparent serious adverse events, while VitD deficiency often occurs among pregnant women. Therefore, several studies have examined how VitD supplementation during pregnancy improves obstetric outcomes [25–27]. Although numerous studies have suggested the benefits of VitD for fertility, some have indicated that excess VitD may have detrimental effects on fertility [28]. Anifandis et al. [29] reported that VitD levels in the follicular fluid are negatively correlated with embryo quality while higher values of VitD are associated with a lower tendency of achieving pregnancy; further, women with overt hypervitaminosis D showed poor IVF outcomes [29]. There is conflicting evidence regarding VitD supplementation and PTB. A recent Cochrane Database Systematic review reported that VitD supplementation may be associated with insignificant or no difference in the risk of PTB at < 37 weeks compared to no intervention or placebo (risk ratio: 0.66, 95% CI: 0.34–1.30; 7 trials, 1640 women, low-certainty evidence) [30]. Conversely, a potential inverse association between maternal VitD status and PTB at < 37 weeks of gestation has been demonstrated [31], which was confirmed by our analysis. Inconsistent with the findings of the Cochrane Database Systematic review, our study showed that higher VitD intake was associated with PTB, especially among ART pregnancies. The mechanisms underlying the increased PTB risk induced by a higher VitD intake remain unclear and speculative. PTB has the same endpoint, consisting of two clinical subtypes—spontaneous PTB, mainly induced by inflammation, and medically-indicated PTB, encountered in cases of SGA or HDP [32]. Higher VitD intake was not associated with either SGA or HDP among ART pregnancies; thus, we considered that the increased risk of PTB among ART pregnancies may be attributed to spontaneous PTB, associated with immune system functioning and inflammation.

Here, the median (interquartile range) pre-pregnancy VitD intake among all participants was 4.3 (2.6–6.7) μg/day. In Japan, the Ministry of Health, Labour and Welfare conducted the National Nutrition and Health survey and reported that the median daily VitD intake among women of 20–49 years was 2.3–2.4 μg/day (number of participants, 823) [33]. An interest in preconception health has recently developed because preconception status markedly influences pregnancy outcomes and the long-term health of both mother and child [10]. Regarding preconception care, appropriate diet counseling may motivate changes in food-intake behavior during pregnancy [1], particularly among women who wish to receive sterility counsel because they have times to consider preconception care [18]. However, no UL exists for pregnant women, and our study indicated that in women who expect to conceive, a higher VitD intake before pregnancy (8.6–12.8 μg/day) may be associated with a shorter gestation period. Therefore, new recommendations regarding VitD intake before pregnancy are required to improve obstetric outcomes.

The major strength of this study is the utilization of data from a large-scale birth cohort study conducted by the Japanese government, with meticulous attention to data collection. Furthermore, this study is considered representative of the general pregnant population in Japan [15]. Additionally, we included a large number of pregnancies achieved either with or without ART. Although a randomized controlled intervention trial is considered a better study design, it is impossible to conduct a long-term controlled trial examining overall dietary intake. Our results were not derived from a randomized controlled study; nevertheless, the large-scale nature of this cohort study allows for the evaluation of potential associations between adverse obstetric outcomes and preconception behaviors [10].

This study has some limitations. First, although we accounted for a few confounding factors, largely covering the questionnaires, other unknown factors may have affected the occurrence of PTB or SGA. Second, subjects were not stratified by the use of specific ART methods such as IVF and/or ICSI or the use of cryopreserved, frozen, or blastocyst embryo transfer. The information for ART also does not include homologous or heterologous. Third, the FFQ used in this study included dietary information for 1 year before pregnancy, but most questions were completed within the first trimester; therefore, a recall bias related to morning sickness may exist. Fourth, the FFQ referred particularly to Japanese alimentary habits and focused on Japanese women. Therefore, our results may not be generalizable to populations of other ethnicities. Fifth, we did not measure serum 25-hydroxy VitD concentrations, which would reflect total VitD in the maternal blood. Additionally, no assessment of VitD intake during pregnancy was performed, which is considered to confer benefits to obstetric outcomes. The absence of this data weakens the argument of an association between higher VitD intake in pregnancy and the subsequent obstetric outcomes. Finally, we did not focus on the effect of daily VitD intake before pregnancy on overall reproductive outcomes because JECS mainly included women who gave live birth.

Growing evidence has suggested the potential advantages of VitD supplementation during pregnancy. Therefore, the significance of sufficient preconception daily VitD intake has been established; however, we indicated that excess VitD intake before pregnancy may affect perinatal outcomes, particularly in women using ART. Women who expect to conceive by ART have more opportunities to undergo preconception care. Both benefits and accumulated toxicity effects of VitD should be considered because it is a fat-soluble vitamin.

Although sufficient daily VitD intake is essential, the results of this study may provide potential harmful effects of excessive intake of daily VitD. The effect of preconception daily VitD intake on obstetric outcomes could depend on the method of conception. We hope that this study will form the basis for appropriate, personalized counseling as a form of preconceptional care for those who will receive ART.

## Data Availability

Data are unsuitable for public deposition due to ethical restrictions and the legal framework of Japan. It is prohibited by the Act on the Protection of Personal Information (Act No. 57 of May 30, 2003, amendment on September 9, 2015) to publicly deposit data containing personal information. Ethical Guidelines for Epidemiological Research enforced by the Japan Ministry of Education, Culture, Sports, Science and Technology and the Ministry of Health, Labour and Welfare also restrict the open sharing of the epidemiologic data. All inquiries about access to data should be sent to: jecs-en@nies.go.jp. The person responsible for handling enquiries sent to this e-mail address is Dr Shoji F. Nakayama, JECS Programme Office, National Institute for Environmental Studies.

## Abbreviations

ART: Assisted reproductive technology
aOR: Adjusted odds ratio
BMI: Body mass index
CI: Confidence interval
FFQ: Food Frequency Questionnaire
HDP: Hypertension disorders of pregnancy
ICSI: Intracytoplasmic sperm injection
IVF: *In vitro* Fertilization
JECS: Japan Environmental Children’s Study
LBW: Low-birth weight
PTB: Preterm birth
SGA: Small for gestational age
UL: Upper intake level
VitD: Vitamin D
